# Potent pro-apoptotic combination therapy is highly effective in a broad range of cancers

**DOI:** 10.1038/s41418-021-00869-x

**Published:** 2021-09-17

**Authors:** Antonella Montinaro, Itziar Areso Zubiaur, Julia Saggau, Anna-Laura Kretz, Rute M. M. Ferreira, Omar Hassan, Ella Kitzig, Ines Müller, Mona A. El-Bahrawy, Silvia von Karstedt, Dagmar Kulms, Gianmaria Liccardi, Johannes Lemke, Henning Walczak

**Affiliations:** 1 Centre for Cell Death, Cancer, and Inflammation (CCCI), UCL Cancer Institute, University College London, 72 Huntley Street, London, WC1E 6DD UK; 2 CECAD Cluster of Excellence, University of Cologne, 50931 Cologne, Germany; 3 Center for Biochemistry, Medical Faculty, Joseph-Stelzmann-Str. 52, University of Cologne, 50931 Cologne, Germany; 4 Department of General and Visceral Surgery, Ulm University Hospital, Albert-Einstein-Allee 23, 89081 Ulm, Germany; 5 Experimental Dermatology, Department of Dermatology, Technical University Dresden, Dresden, Germany; 6 Department of Histopathology, Imperial College London, London, W12 0NN UK; 7 Department of Translational Genomics, Center of Integrated Oncology Cologne-Bonn, Medical Faculty, University of Cologne, 50931 Cologne, Germany; 8 Center for Molecular Medicine Cologne, Medical Faculty, University Hospital of Cologne, 50931 Cologne, Germany

**Keywords:** Cancer models, Cell biology, Preclinical research

## Abstract

Primary or acquired therapy resistance is a major obstacle to the effective treatment of cancer. Resistance to apoptosis has long been thought to contribute to therapy resistance. We show here that recombinant TRAIL and CDK9 inhibition cooperate in killing cells derived from a broad range of cancers, importantly without inducing detectable adverse events. Remarkably, the combination of TRAIL with CDK9 inhibition was also highly effective on cancers resistant to both, standard-of-care chemotherapy and various targeted therapeutic approaches. Dynamic BH3 profiling revealed that, mechanistically, combining TRAIL with CDK9 inhibition induced a drastic increase in the mitochondrial priming of cancer cells. Intriguingly, this increase occurred irrespective of whether the cancer cells were sensitive or resistant to chemo- or targeted therapy. We conclude that this pro-apoptotic combination therapy has the potential to serve as a highly effective new treatment option for a variety of different cancers. Notably, this includes cancers that are resistant to currently available treatment modalities.

## Introduction

Chemotherapy and/or radiotherapy still represent the first line of treatment for the majority of cancer patients. However, most patients develop resistance to these therapeutic modalities, ultimately leading to disease progression [1]. The identification of specific oncogenic drivers and the development of inhibitors to target them has resulted in prolonged patient survival in several cancer entities [2, 3]. However, despite the undoubted success of certain targeted therapeutic approaches [4], unfortunately they often provide benefit only to a limited number of patients [5] and, almost inevitably, patients develop resistance also to these targeted therapies [6, 7].

Inactivation of the apoptosis pathway is a major contributor to drug resistance [8, 9]. For the past three decades, an orthogonal approach to the development of inhibitors of oncogenic drivers has been the study of how to best harness the cell’s machinery to undergo apoptosis to specifically and highly effectively kill cancer cells with the aim to develop a more effective means to treat cancer [10, 11]. During the past decade this research has resulted in the development of highly potent treatments for certain lymphoid and myeloid malignancies, achieved by pro-apoptotic perturbation of the mitochondrial arm of the apoptosis pathway through inhibition of Bcl-2 [12–15]. However, the targeting of the death receptor arm of the apoptosis pathway has yet to prove its usefulness in the cancer clinic [16–18].

Several members of the tumor necrosis factor (TNF) superfamily of cytokines are naturally expressed by activated immune cells and involved in the killing of target cells. These so-called death ligands have been particularly attractive as potential novel cancer therapeutics. Yet, TNF and Fas ligand (FasL; also known as CD95L) both induced lethal toxicities when applied systemically [19–21] which prevented their respective use as anticancer therapeutics. This was different for the TNF-related apoptosis-inducing ligand (TRAIL; also known as Apo2L) as it was found to selectively induce apoptosis in cancer cells, yet not in any essential normal cells in vitro and in vivo [22, 23].

TRAIL binding to the its membrane-bound receptors TRAIL-R1 (DR4) [24] and TRAIL-R2 (DR5) [25, 26] induces formation of the death-inducing-signaling complex (DISC) and, thereby, the activation of caspases 8 and 10 and the intracellular apoptosis signaling cascade [27, 28]. Apart from a recombinant form of TRAIL, various other TRAIL-R agonists have been developed for clinical use [16]. However, clinical testing of these first generation TRAIL-R agonists did not reveal meaningful anticancer activity in patients, likely owed to the combination of their limited agonistic activity [22, 29] and the fact that most cancers indeed exhibit primary resistance to apoptosis induction by TRAIL or other TRAIL-R agonists when used as single agents [16, 17, 29, 30]. Therefore, numerous studies have been undertaken with the aim of breaking cancer cell resistance to TRAIL-induced apoptosis and a plethora of combination treatment strategies to therapeutically overcome this resistance have been proposed [17, 30, 31]. Amongst the most promising of the previously identified means to achieve this are combinations of TRAIL with inhibitors of the proteasome [32, 33], antagonists of inhibitor of apoptosis (IAP) proteins [29, 34], or inhibitors of cyclin-dependent kinase 9 (CDK9) [35], with the latter being the most potent TRAIL sensitization strategy identified to date [17, 30].

The experiments in the latter study combining TRAIL with CDK9 inhibition (TRAIL–CDK9i) were performed on a panel of non-small cell lung cancer (NSCLC) cell lines [35]. However, two major questions remained unanswered: (i) does the high potency of this combination treatment extend to cancer entities beyond NSCLC and (ii) could this combination therapy also serve to effectively kill cancer cells with primary or acquired resistance to chemo- or targeted therapy, the major obstacle to successful cancer therapy? Here we show that TRAIL–CDK9i exerts high potency and broad applicability across many cancer types, indeed all we tested thus far. Importantly, high therapeutic efficacy is achieved in the absence of detectable, let alone prohibitive untoward effects in vivo. Most strikingly, however, regardless of the type of cancer tested, TRAIL–CDK9i is also capable of effectively killing cancer cells with primary or acquired resistance to chemo- or targeted therapy. Mechanistically, our results suggest that the high efficacy of this combination treatment relies on the ability of CDK9i to downregulate cFLIP and Mcl-1, which respectively facilitate the ability of TRAIL to induce caspase-8-mediated Bid cleavage and tBid-mediated activation of Bax/Bak to induce mitochondrial permeabilization and consequent apoptosis. We conclude that the TRAIL–CDK9i therapeutic regime may serve as an effective treatment alternative for a wide range of cancer patients.

## Results

### TRAIL–CDK9i combination therapy is broadly applicable and more efficacious than standard-of-care therapies

We first sought to evaluate whether the efficacy of the TRAIL–CDK9i combination we previously observed on a panel of NSCLC cell lines [35] was cancer entity-selective or more widely applicable. To target CDK9 we employed dinaciclib, the clinically most advanced CDK9-targeting drug [36], and, in parallel, NVP-2, a recently derived highly selective and specific CDK9 inhibitor [37]. In either case, their respective CDK9-inhibitory activity was both, required and sufficient for their capacity to sensitize cancer cells to TRAIL-induced apoptosis (Supplementary Fig. 1a, b).

**Fig. 1:**
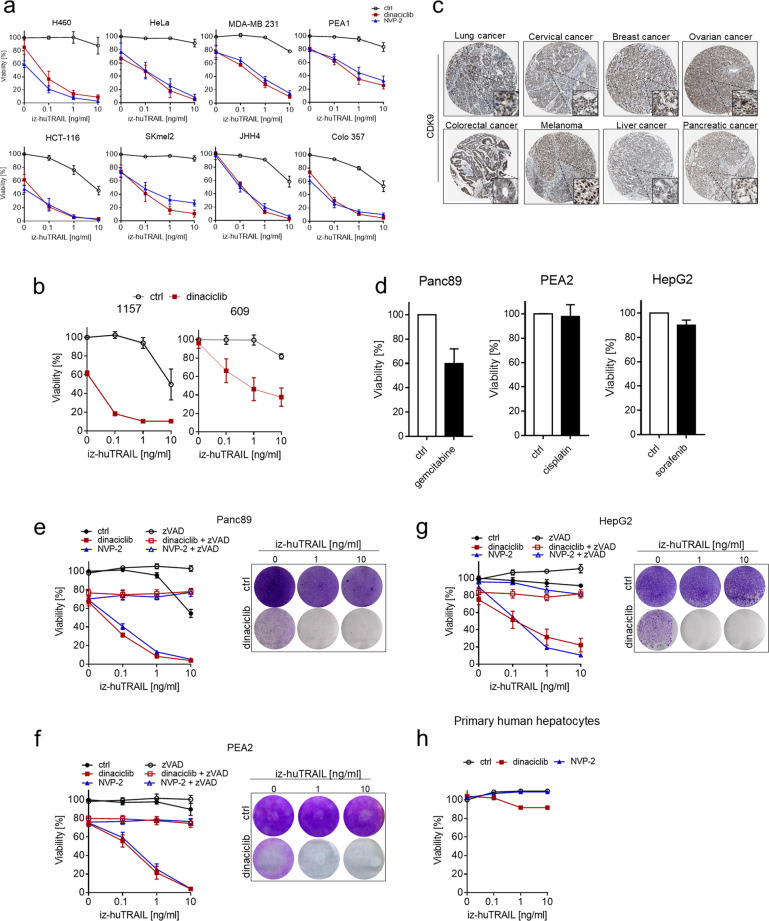
TRAIL-CDK9i is broadly applicable and significantly more efficacious than current first-line treatments for different solid cancers. **a** A panel of human carcinoma cell lines was treated with DMSO, dinaciclib (25 nM) or NVP-2 (25 nM) for 1 h before iz-huTRAIL was added at the indicated concentrations and the cells were incubated for an additional 24 h. Cell viability was determined by CellTiter-Glo. **b** Primary human PDAC cancer cells were preincubated with DMSO or dinaciclib (25 nM) for 1 h before iz-huTRAIL was added at the indicated concentrations. Cell viability was quantified after 24 h. **c** CDK9 protein expression summary from the Human Protein Atlas (www.proteinatlas.org). **d** Pancreatic (Panc89), ovarian (PEA2) and liver (HepG2) cancer cells were treated for 48 h with DMSO, gemcitabine (1 µM), cisplatin (1 µM) or sorafenib (7 µM) cell viability was determined by CellTiter-Glo. **e** Panc89, **f** PEA2 and **g** HepG2 cancer cells were pre-treated with DMSO, dinaciclib (25 nM) or NVP-2 (25 nM) ± zVAD (20 μM) for 1 h before iz-huTRAIL was added at the indicated concentrations and the cells were incubated for an additional 24 h. Cell viability was determined as above. The right panels indicate cell lines pre-treated with DMSO or dinaciclib (25 nM) for 1 h and subsequently stimulated with iz-huTRAIL at the indicated concentrations for 24 h. Long-term survival was visualized after 7 days by crystal violet staining. One of three independent experiments is shown. **h** Primary human hepatocytes were pre-treated with DMSO, dinaciclib (25 nM) or NVP-2 (25 nM) for 1 h before iz-huTRAIL was added at the indicated concentrations and the cells were incubated for an additional 24 h. Cell viability was determined by CellTiter-Glo. Data are mean ± SEM, *n* ≥ 3.

We therefore treated several cell lines representing various cancer entities, including, lung, cervical, breast, ovarian, colorectal, liver and pancreatic cancer with TRAIL and/or CDK9i (Supplementary Fig. 1c). Strikingly, CDK9i consistently exerted potent sensitization of these different cancer cell lines to TRAIL-induced cell death (Fig. 1a and Supplementary Fig. 1d). Importantly, TRAIL–CDK9i was also highly potent at killing primary patient-derived human pancreatic ductal adenocarcinoma (PDAC) cells (Fig. 1b). Interestingly, across several cancer types the expression levels of CDK9 protein, as analyzed by immunohistochemistry in the human protein pathology atlas [38], confirmed high CDK9 expression in diverse tumor types (Fig. 1c and Supplementary Fig. 1e). Thus, CDK9 is highly expressed in all cancer entities tested and its inhibition represents a valid approach which appears to broadly sensitize tumor cells to TRAIL-induced cell death.

Given the immense challenge to identify novel therapeutic strategies which could offer treatment alternatives that are significantly more efficacious than current standard-of-care therapies, we treated several cancer cell lines which showed only a partial or low response to their respective first-line treatments (Fig. 1d and Supplementary Fig. 1f) with TRAIL and/or the CDK9 inhibitor. Invariably, this revealed that CDK9i exerted potent sensitization of these different cancer cell lines to TRAIL-induced apoptosis and that TRAIL–CDK9i combination treatment was substantially more effective at killing these cells than their respective currently applied stand-of-care therapy (Fig. 1e-g and Supplementary Fig. 1f).

Two major risks of potentially highly effective new cancer treatment opportunities identified through in vitro strategies are (i) the rapid selection for cellular resistance and (ii) toxicity. To address the potential risk of selection for therapy resistance, we studied the effect of TRAIL–CDK9i on clonogenic cancer cell outgrowth. TRAIL–CDK9i completely or nearly completely abolished clonogenic cell survival in cell lines representing all cancer entities tested, including when TRAIL was applied at low concentrations (1–10 ng/ml) and for as short as 24 h (Fig. 1e-g, right panels). Importantly, primary human hepatocytes were not killed by TRAIL–CDK9i when TRAIL was used at these concentrations (Fig. 1h). Together, these results demonstrate that TRAIL–CDK9i is more effective than current standard-of-care treatments and it is capable of inducing apoptosis with high efficacy, yet in a cancer-selective manner, in a wide range of human cancer cell lines representing various tumor entities, including PDAC.

### TRAIL–CDK9i is more effective than the current standard-of-care in an in vivo model of pancreatic cancer

**Fig. 2:**
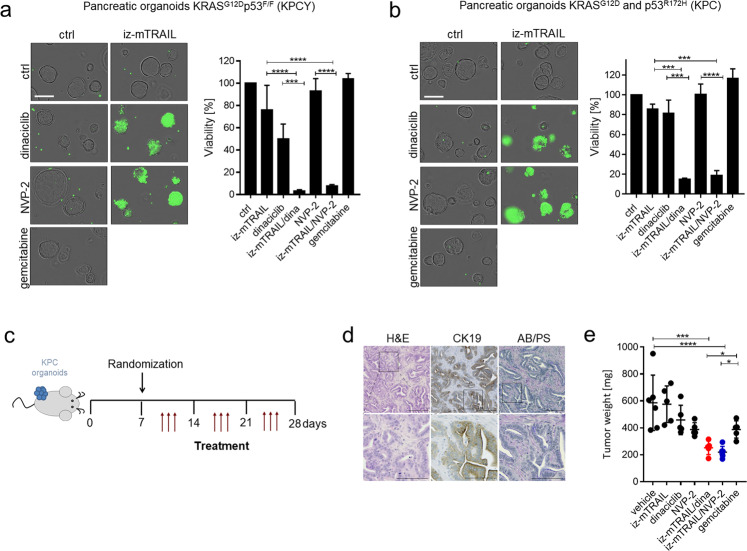
TRAIL combined with CDK9 inhibition shows therapeutic efficacy in pancreatic organoids in vitro and in vivo. **a**, **b** Sytox-positive KPCY or KPC organoids were treated for 24 h with DMSO, iz-mTRAIL (1000 ng/ml) and/or dinaciclib (100 nM) and/or NVP-2 (100 nM) or with gemcitabine (1 μM). Scale bars, 200 µm (left panels). Cell viability of KPCY or KPC organoids was determined by CellTiter-Glo (right panels). Data are mean ± SEM, *n* ≥ 3. **c** Experimental protocol: iz-mTRAIL (5 mg/kg) and/or dinaciclib (30 mg/kg); and/or NVP-2 (5 mg/kg) or gemcitabine (50 mg/kg) or 200 µl buffer were administered intraperitoneally 7 days after subcutaneous injection of 1 × 10^6^ KPC cells. **d** Histological analysis of KPC tumors and corresponding CK19 and AB/PAS stain. Black box indicates region enlarged on the bottom. **e** Tumor weight quantification at day 28. Dots represent individual mice. Error bars represent mean ± SEM.

Because PDAC is often diagnosed at an advanced stage and because it frequently exhibits primary resistance to gemcitabine, the current first-line therapy, PDAC remains one of the most lethal human cancers [39]. To determine whether TRAIL–CDK9i may be effective at killing cancer cells in a clinically relevant experimental model of PDAC, we made use of organoid cultures which closely resemble the three-dimensional in vivo structure of PDAC tumors [40]. We first tested PDAC organoids derived from *Kras*^*LSL-G12D/WT*^*; p53*^*LSL-R172H/WT*^*; Pdx-Cre* (KPC) mice and *KRas*^*LSL-G12D/WT*^*; P53*^*F/F*^*; Pdx-Cre*; *Rosa26-LSL-YFP* (KPCY) mice for sensitivity to TRAIL–CDK9i as compared to the standard-of-care treatment with gemcitabine in vitro. Whilst unsurprisingly exhibiting high resistance to gemcitabine, these PDAC organoids were exquisitely sensitive to killing by recombinant murine TRAIL (mTRAIL) in combination with dinaciclib or NVP-2 but not by any of these three drugs alone (Fig. 2a, b).

We next assessed the therapeutic efficacy of TRAIL–CDK9i in comparison to gemcitabine on KPC-derived organoids in vivo. One week after subcutaneous injection of KPC-derived organoids into immunocompetent C57BL/6 mice (Fig. 2c and Supplementary Fig. 2), the mice developed subcutaneous tumors characterized by cytokeratin 19 (CK19)-expressing ductal networks and the presence of AB/PAS-positive lesions, two hallmark characteristics of pancreatic intraepithelial neoplasias (PanINs) (Fig. 2d). These PDAC precursor lesions were largely resistant to treatment by gemcitabine, mTRAIL, dinaciclib, or NVP-2 alone. However, both mTRAIL–CDK9i combination treatments, i.e., mTRAIL–dinaciclib and mTRAIL–NVP-2, achieved a highly significant in vivo anti-tumor effect (Fig. 2e). These results suggest that combining a TRAIL-R agonist with a CDK9-inhibiting drug could prove substantially more effective in the treatment of PDAC than the current first-line treatment for this type of cancer.

### TRAIL–CDK9i induces remarkable therapeutic anti-tumor efficacy in vivo without causing systemic toxicity

**Fig. 3:**
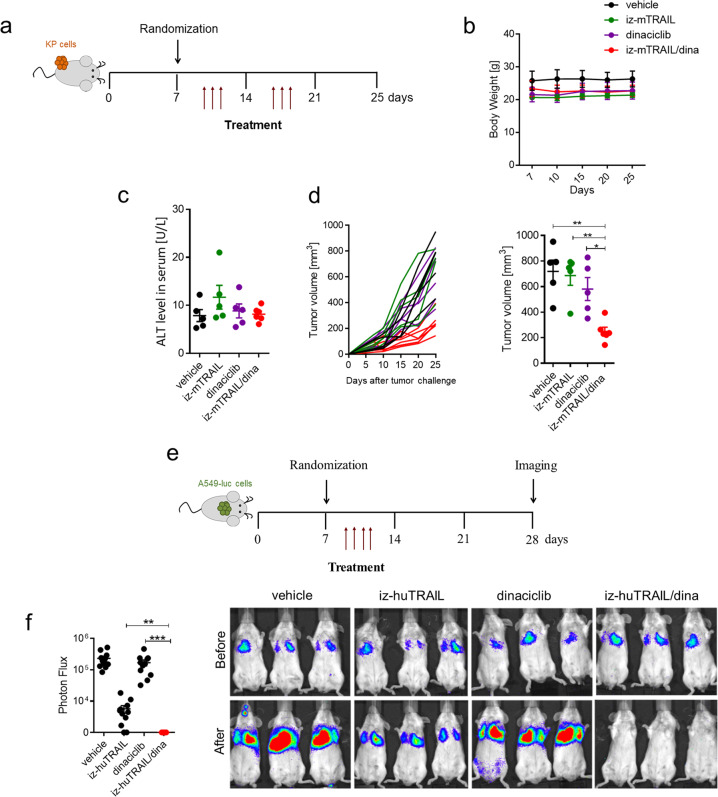
Co-treatment with TRAIL and CDK9i is well tolerated and exerts therapeutic efficacy in vivo. **a** Experimental protocol: iz-mTRAIL (5 mg/kg) and/or dinaciclib (30 mg/kg) or 200 µl buffer were administered intraperitoneally 7 days after subcutaneous injection of 0.5 × 10^6^ KP cells. **b** Average body weight of KP-bearing mice measured weekly from baseline to 1 week after the end of drug administration. **c** Serum ALT value 24 h after the last drug administration. **d** Growth curves of individual KP tumors are shown (left graph) and tumor volume quantification at day 25 (right graph). Dots represent individual mice (*n* = 5 per group). Error bars represent mean ± SEM. **e** Experimental protocol: severe combined immunodeficiency (Scid beige) mice were injected with 2 × 10^6^ A549 cells stably expressing luciferase into the lateral tail vein. Seven days after tumor inoculation mice were treated i.p. with iz-huTRAIL (5 mg/kg) and/or dinaciclib (30 mg/kg) or with 200 µl buffer as control for four consecutive days. **f** Tumor burden was assessed after 28 days via bioluminescence imaging. Dots represent individual mice (*n* = 11 per group). Error bars represent mean ± SEM.

Apart from preclinical in vivo assessment of efficacy of novel cancer therapies, also the determination of potential toxicities they may induce is crucial to assess their clinical applicability, especially when a member of the death ligand family is concerned [20, 21, 41, 42]. We therefore next assessed both, therapeutic efficacy and, in parallel, potential toxicities exerted by the mTRAIL–CDK9i combination. To do so, we intradermally injected murine NSCLC cells derived from KP mice (Kras^LSL-G12D/WT^; p53^F/F^) (Supplementary Fig. 3a) into recipient syngeneic mice and, following establishment of tumors (Supplementary Fig. 3b), treated tumor-bearing mice systemically every other day for two consecutive weeks with vehicle as a control or with mTRAIL and/or dinaciclib (Fig. 3a). This revealed that the combination of mTRAIL with dinaciclib was well tolerated by the mice as we could neither observe changes to body weight (Fig. 3b) nor significant differences in serum levels of Alanine transaminase (ALT) between treatment groups (Fig. 3c). Of note, whilst single-agent therapy with mTRAIL or dinaciclib was virtually ineffective (Fig. 3d), the combination of mTRAIL with dinaciclib achieved remarkable anti-tumor efficacy (Fig. 3d). We therefore conclude that treatments combining a TRAIL-R agonist with a CDK9-inhibiting drug can achieve high anticancer efficacy in vivo, importantly without causing detectable adverse events.

To further evaluate the efficacy of TRAIL–CDK9i in vivo we injected stably luciferase-expressing A549 cells (A549-luc) into the tail vein of host mice, resulting in the development of lung tumors in situ seven days after tumor cell injection, as determined by in vivo bioluminescence imaging (Fig. 3e and Supplementary Fig. 3c). Whilst TRAIL–CDK9i treatment achieved complete lung tumor regression by day 28 (Fig. 3f and Supplementary Fig. 3d), this was not the case with TRAIL or CDK9i treatment alone, further supporting the high synergy and efficacy of this treatment combination in vivo.

### TRAIL–CDK9i is highly effective at killing chemo- and targeted therapy-resistant cancer cells, independently of the resistance-causing agent

Acquired resistance to the different modalities of cancer therapy, including to chemo-, radio- or targeted therapies and, more recently, also to immune checkpoint blockade, remains the major obstacle to effective cancer therapy [6, 43]. Consequently, achieving significant improvement in the survival of cancer patients would require the identification of treatment strategies that can either overcome or, alternatively, bypass the current major impasse of therapy resistance, if possible, to more than one of the afore-mentioned treatment modalities simultaneously. Encouraged by the results obtained with TRAIL–CDK9i thus far, including on intrinsically chemotherapy-resistant PDAC (Fig. 2a–e), we next enquired whether this combination therapy may afford the opportunity to effectively kill cancers resistant to current therapies and, if so, how broadly this may apply. To address this question, we generated therapy-resistant versions of cancer cell lines derived from several distinct cancer entities. Whereas some of these cell lines were rendered resistant to chemotherapy, others represent resistance to various distinct types of targeted therapies.

**Fig. 4:**
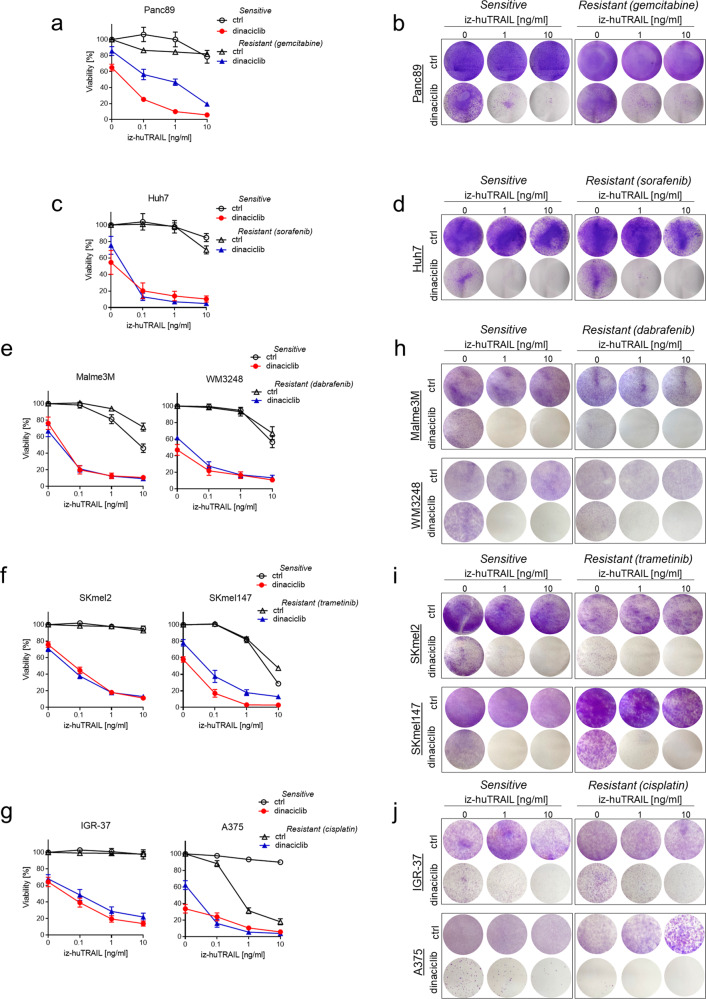
TRAIL–CDK9i cooperate in killing chemo- and to targeted therapies-resistant cells. **a, b** Gemcitabine-sensitive or -resistant pancreatic cancer cells and **c**, **d** and sorafenib-sensitive or -resistant hepatocellular carcinoma cells were pre-incubated with DMSO or dinaciclib (25 nM) for 1 h and subsequently treated with iz-huTRAIL at the indicated concentrations. Cell viability was quantified after 24 h (left panels). Long-term survival was visualized after 7 days by crystal violet staining (right panels). **e–j** Treatment of chemo- and targeted therapy sensitive versus resistant melanoma cells: dabrafenib-sensitive and -resistant (**e** and **h**), trametinib-sensitive and -resistant (**f** and **i**) and cisplatin-sensitive and -resistant (**g** and **j**) melanoma cells were pre-incubated with DMSO or dinaciclib (25 nM) for 1 h and subsequently treated with iz-huTRAIL at the indicated concentrations. Cell viability was quantified after 24 h (left panels). Data are mean ± SEM, *n* ≥ 3. Long-term survival was visualized after 7 days by crystal violet staining (right panels). One representative of three independent experiments is shown.

To assess the capacity of TRAIL–CDK9i to cooperate in killing chemotherapy-resistant cells, we established a gemcitabine-resistant variant of the human PDAC cell line Panc89 (Supplementary Fig. 4a). Gemcitabine-resistant Panc89 cells retained the high sensitivity of the parental Panc89 cells to TRAIL–CDK9i, whereas neither TRAIL nor CDK9i alone exerted any significant effects on cancer cell survival (Fig. 4a). Of note, also the clonogenic capacity of Panc89 cells was drastically reduced by TRAIL–CDK9i. Intriguingly, this was irrespective of gemcitabine sensitivity or resistance, implying that TRAIL–CDK9i effectively interferes with the survival of clones even if they have evolved to acquire resistance to gemcitabine (Fig. 4b). To assess whether TRAIL–CDK9i would also be effective at killing cells resistant to sorafenib, a targeted therapeutic which is employed as first-line treatment of hepatocellular carcinoma (HCC), we next derived sorafenib-resistant Huh7 HCC cells (Supplementary Fig. 4b). Again, TRAIL–CDK9i killed both, parental and sorafenib-resistant Huh7 cells with high efficacy (Fig. 4c, d).

Despite the fact that various new forms of targeted therapies as well as immune checkpoint blockade have been added to the clinical armamentarium of melanoma treatment over the past decade, melanoma remains a hard-to-treat cancer, not the least because development of resistance to these novel therapeutic approaches has become a major current clinical challenge in the treatment of this malignancy [44]. We, therefore, next tested whether TRAIL–CDK9i were able to kill melanoma cells that have developed resistance to chemo- or targeted therapy and whether or not such an effect, should we observe it, depended on the nature of the resistance-causing agent. Using the BRAF inhibitor dabrafenib, the MEK inhibitor trametinib and the chemotherapeutic agent cisplatin, we first derived a panel of melanoma cell lines that respectively developed resistance to these three therapeutics (Supplementary Fig. 4c, d). We subsequently treated these cell lines and their respective corresponding therapy-sensitive parental counterparts with TRAIL, CDK9i or the TRAIL–CDK9i combination. Strikingly, only TRAIL–CDK9i, but neither TRAIL nor CDK9i alone, was highly effective at killing all parental as well as all therapy-resistant melanoma cell lines tested, importantly regardless of the nature of both, their respective oncogenic driver mutations and their respective acquired chemo- or targeted therapy resistance (Fig. 4e-g). When examining the effect of the combination therapy on clonal survival of these melanoma cells, we observed that TRAIL–CDK9i entirely repressed the capacity of clonal outgrowth in both, therapy-sensitive and -resistant melanoma cells (Fig. 4h-j). We, therefore, conclude that the combination of a TRAIL-receptor agonist with a CDK9-inhibiting drug could potentially serve as an effective therapy for various types of cancers that have developed resistance to current therapies.

### TRAIL–CDK9i combination treatment increases the apoptotic priming of cancer cells

**Fig. 5:**
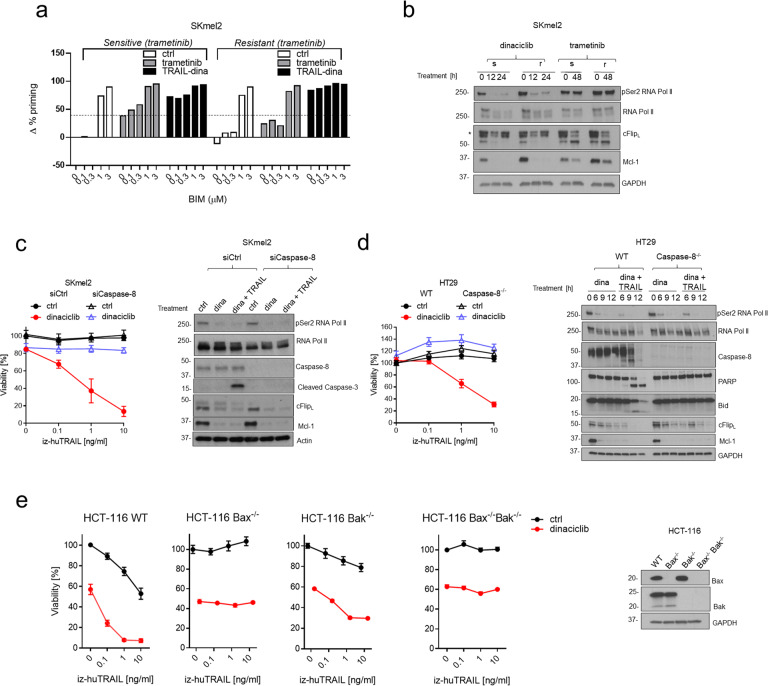
Molecular mechanisms of sensitization to TRAIL–CDK9i combination. **a** Dynamic BH3 profiling was performed on trametinib-sensitive and -resistant SKmel2 cells following treatment for 48 h with trametinib (10 nM) or for 12 h with a combination of dinaciclib (25 nM) and TRAIL (10 ng/ml). The Bim-derived BH3-only peptide was employed at different concentrations (0.1–3.0 μM) to assess mitochondrial apoptotic priming status. Results are expressed as Δ% priming (increase in priming compared to untreated cells). Data are mean ± SEM, *n* ≥ 3. **b** Trametinib-sensitive and -resistant SKmel2 cells were treated with trametinib (10 nM) or with dinaciclib (25 nM) for the indicated times. Cells were lysed and subjected to western blotting with antibodies specific for the indicated antigens. One representative of two independent experiments is shown. An asterisk indicates an unspecific band. **c** SKmel2 cells were pre-incubated with DMSO or dinaciclib (25 nM) for 1 h and subsequently treated with iz-huTRAIL at the indicated concentrations. Cell viability was quantified after 24 h (left panel). Data are mean ± SEM, *n* ≥ 3. SKmel2 cells were pretreated with DMSO or dinaciclib (25 nM) for 4 h and subsequently stimulated with iz-huTRAIL (10 ng/ml) for 6 h. Cells were lysed and subjected to western blotting. One representative of two independent experiments is shown (right panel). **d** HT29 cells were pre-incubated with DMSO or dinaciclib (25 nM) for 1 h and subsequently treated with iz-huTRAIL at the indicated concentrations. Cell viability was quantified after 24 h (left panel). Data are mean ± SEM, *n* ≥ 3. HT29 cells were pretreated with DMSO or dinaciclib (25 nM) for 3 h and subsequently stimulated with iz-huTRAIL (10 ng/ml) for the indicated times. Cells were lysed and subjected to western blotting (right panel). One representative of two independent experiments is shown. **e** HCT-116 wild-type (WT), HCT-116 Bax^−/−^, HCT-116 Bak^−/−^ or HCT-116 Bax^−/−^Bak^−/−^ cells were pre-incubated with DMSO or dinaciclib (25 nM) for 1 h and subsequently treated with iz-huTRAIL at the indicated concentrations. Cell viability was quantified after 24 h (left panels). Data are mean ± SEM, *n* ≥ 3. Representative western blots of knockout efficiency are shown (right panel).

We next aimed to uncover the mechanism by which TRAIL–CDK9i achieved to induce apoptosis with such efficacy in melanoma cells that had been rendered resistant to either chemo- or targeted therapy. To do so, we employed dynamic BH3 profiling which determines early changes in pro-apoptotic signaling at the level of mitochondrial ‘priming’ in response to diverse apoptotic stimuli in cancer cells and, thereby, serves as a predictor of a particular therapy’s cytotoxic capacity [45]. Using increasing concentrations of the Bcl-2 homology domain 3 (BH3) peptide of the BH3-only protein Bim (0.1–3.0 μM) showed that chemo- and targeted therapy-resistant melanoma cells did not grossly differ in their basal priming status (Fig. 5a and Supplementary Fig. 5a–c). However, when treated with chemo- or targeted therapeutics, therapy-resistant cell lines required the Bim peptide at a concentration of least 1 μM to achieve apoptotic priming whereas in their sensitive counterpart apoptotic priming was already apparent at 100–300 nM (Fig. 5a and Supplementary Fig. 5a–c). By contrast, TRAIL–CDK9i drastically enhanced mitochondrial depolarization, strikingly achieving it even in the absence of exogenously added Bim peptide in all six melanoma cell lines, representing the three chemo-/targeted therapy-sensitive and -resistant melanoma cell line pairs (Fig. 5a and Supplementary Fig. 5a–c). These results show that the combination of TRAIL with CDK9i is capable of drastically lowering the apoptotic threshold in cancer cells, importantly, including in therapy-resistant cancer cells.

We previously showed that downregulation of both, cFLIP and Mcl-1 are required for CDK9i-mediated sensitization to TRAIL-induced apoptosis [35]. We, therefore, next analyzed the expression levels of the different anti-apoptotic components of the TRAIL apoptosis pathway in therapy-sensitive as well as -resistant cancer cells. This showed that CDK9i efficiently suppressed the levels of Mcl-1 and cFLIP not only in therapy-sensitive but, importantly, also in therapy-resistant melanoma cells (Fig. 5b and Supplementary Fig. 6a-b). Moreover, whilst downregulation of cFLIP and Mcl-1 readily occurred in caspase-8-deficient cancer cells, absence of caspase-8 completely prevented the TRAIL-dependent appearance of pro-apoptotic tBid, cleaved poly-ADP ribose polymerase (PARP) and cleaved caspase-3 which rendered these cells resistant to apoptosis induction by TRAIL–CDK9i (Fig. 5c-d). In addition, inhibition of the activation of the mitochondrial apoptosis pathway by deletion of Bax and/or Bak also rendered cancer cells resistant to CDK9i-mediated sensitization to TRAIL-induced cell death (Fig. 5e). Together, these results show that CDK9i-mediated TRAIL sensitization relies on the downregulation of the anti-apoptotic factors cFLIP and Mcl-1 and on the activities of caspase-8 and Bax/Bak.

### TRAIL–CDK9i sensitizes colorectal cancer patient-derived organoids to TRAIL-induced cell death

**Fig. 6:**
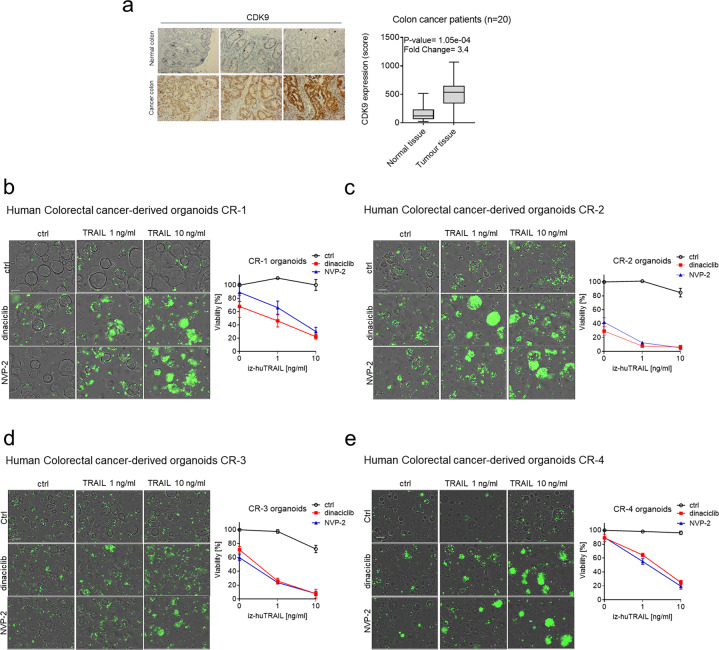
TRAIL–CDK9i is highly effective at killing colorectal cancer patient-derived organoids. **a** Comparing protein expression analysis on tumor and normal tissues of 20 colorectal patients revealed significantly increased expression of CDK9 in tumor versus normal colorectal tissue. Representative pictures of immunohistochemically determined CDK9 expression on normal human colon tissue and colorectal cancer tissues (left panel). Statistical significance between groups was determined using Wilcoxon signed-rank test for group comparison demonstrated by boxplot. *P* values of <0.05 were considered statistically significant. **b–e** Sytox-positive colorectal cancer-derived organoids were treated for 24 h with DMSO, iz-TRAIL (0–10 ng/ml) and/or dinaciclib (25 nM) and/or NVP-2 (25 nM). Scale bars, 200 µm (left panels). Cell viability was quantified after 24 h (right panels). Data are mean ± SEM, *n* ≥ 3.

Another hard-to-treat cancer is colorectal carcinoma (CRC). An analysis of 20 pairs of CRC and adjacent non-tumor colon tissue by immunohistochemistry revealed that CDK9 expression was increased significantly in tumor versus non-tumor tissue, suggesting CDK9 as a potentially suitable therapeutic target also in this large tumor entity (Fig. 6a). Patient-derived organoids, cultured and treated in three-dimensional systems, are widely regarded as reliably modeling the clinical response of individual patients to therapy [46]. We, therefore, next examined a panel of CRC patient-derived organoids which we cultured in a three-dimensional system for susceptibility to the TRAIL–CDK9i combination therapy. Strikingly, CDK9i markedly sensitized all four CRC patient-derived organoids we tested to TRAIL-induced cell death (Fig. 6b–e). We therefore conclude that the TRAIL–CDK9i treatment combination may also prove efficacious in the treatment of CRC.

## Discussion

Therapy resistance, both primary and acquired, remains the major obstacle to effective cancer therapy [47, 48]. Taking an orthogonal approach to the current major efforts in cancer research towards more personalized treatments through the targeting of specific oncogenic driver mutations, we have investigated how we can best harness the cell’s machinery to undergo programmed cell death by apoptosis for the specific and highly effective killing of cancer cells with the aim of identifying therapies which may prove highly efficacious for a wide range of cancers. We here identify the combination of the specific inhibition of the kinase activity of CDK9 with the agonistic activation of TRAIL death receptors as a highly effective means to induce apoptosis across a wide range of cancer entities. Importantly, regardless of tumor type, TRAIL–CDK9i combination therapy also effectively eliminated cancer cells with primary or acquired resistance to different forms of standard-of-care therapies. Strikingly, the TRAIL–CDK9i combination also proved highly effective in cancer patient-derived organoids as well as in syngeneic cancers in vivo, importantly without causing prohibitive or noticeable untoward effects.

We further demonstrate that murine PDAC organoids, driven by *KRAS* mutation in combination with *TP53* mutation or loss of expression, which were largely resistant to chemotherapy, were highly sensitive to the TRAIL–CDK9i combination in vitro and in vivo. Given the remarkable therapeutic efficacy we observed with the TRAIL–CDK9i combination in various models of hard-to-treat cancers, one may envisage that this treatment combination may become widely applicable in cancer therapy, including in the treatment of KRAS-mutated cancers.

In line with the idea that cancer cells require increased transcriptional elongation for their high turn-over metabolism, CDK9 has been shown to be dysregulated in several cancers [49–51] to favor cancer cell proliferation, apoptosis resistance and spread. We recently showed that high CDK9 expression in pancreatic tumor tissue is associated with significantly shortened survival [52]. Thus, high CDK9 expression may serve as a marker to identify patients who may particularly benefit from treatment with CDK9i and, according to the results presented here, especially the TRAIL–CDK9i combination.

We show here that TRAIL–CDK9i is capable of reducing the threshold for induction of mitochondrial apoptosis so potently that cancer cells which had been rendered resistant to chemo- or targeted therapy were still readily killed by TRAIL–CDK9i with high efficacy. Mechanistically, we found that CDK9i simultaneously removes Mcl-1 and cFLIP, importantly from both chemo- and targeted therapy-sensitive as well as -resistant cell lines. The role of Mcl-1 is to bind and thereby inhibit pro-apoptotic Bcl-2 family members such as Bim, PUMA, NOXA, and tBid, whereas the role of cFLIP is to prevent caspase-8 from cleaving substrates, including Bid, in the context of death receptor–ligand signaling, including when triggered by TRAIL. Here we show that caspase-8 is required for TRAIL–CDK9i-induced generation of tBid and that both caspase-8 and Bax/Bak are required for TRAIL–CDK9i-induced apoptosis. Together, these results suggest the following mechanism for the TRAIL–CDK9i-mediated reduction in threshold for mitochondrial priming and consequent apoptosis induction: (i) CDK9i-mediated cFLIP downregulation facilitates the capacity of TRAIL to activate caspase-8; (ii) activated caspase-8 cleaves Bid to generate pro-apoptotic tBid; (iii) the pro-apoptotic tBid generated thereby, in combination with the CDK9i-mediated downregulation of Mcl-1, enables activation of Bax and Bak on mitochondria and, consequently, the highly effective induction of apoptosis by TRAIL–CDK9i.

Based thereupon, we propose the combination of TRAIL receptor agonists with drugs capable of inhibiting CDK9 for clinical evaluation in a wide variety of cancers. Our results suggest that this should include cancers that are resistant to currently available therapies, offering the opportunity to tackle a major current obstacle to the successful treatment of cancer.

## Materials and methods

### Data reporting

No statistical methods were used to predetermine sample size.

### Cell lines

The human lung adenocarcinoma cell lines Calu-1, H460, and H322 were kindly provided by J. Downward, A549-luc cells were purchased from Caliper Life Science and cultured in RPMI (Gibco™, Thermo Fisher Scientific) supplemented with 10% Fetal Calf Serum (FCS). Colo357 cells were kindly provided by A. Trauzold, HCT116 wild-type, Bak^-/-^, Bax^-/-^ and Bax^-/-^Bak^-/-^ cells were kindly provided by A. J. García-Sáez, HT29 Caspase-8^-/-^ were kindly provided by M. Pasparakis, all cells were cultured in DMEM (Gibco™, Thermo Fisher Scientific) supplemented with 10% FCS. All ovarian cancer cell lines were cultured in RPMI 1640 supplemented with 10% FCS and 1% penicillin/streptomycin/glutamine (10,000 U/ml, Gibco™, Thermo Fisher Scientific). HeLa, MDA-MB 231 and all human liver and pancreas carcinoma cells were cultured in DMEM supplemented with 10% FCS. Primary human hepatocytes were purchased from Gibco/Invitrogen (Paisley, UK) and cultured according to the manufacturer’s instructions. Melanoma cell lines were cultured in RPMI 1640 supplemented with 10% FCS. The resistant cell lines were generated by continuous treatment with the indicated drugs for at least 6 months. All cell lines were mycoplasma-free as determined by *MycoAlert*™ Mycoplasma Detection Kit (LONZA). Primary PDAC cell lines 609 and 1157 were kindly provided by A. Liss and were previously established from xenograft tumors derived from surgically resected PDAC [53]. The cell lines were cultured in DMEM/F12 medium complemented with 10% fetal bovine serum (Biochrom, Merck) and 1% penicillin-streptomycin at 37 °C and 5% CO_2_.

### Pancreatic tumor cell isolation and organoid cultures

KPCY organoids were derived from PDAC that developed in *KRas*^*LSL-G12D/WT*^*; P53*^*F/F*^*; Pdx-Cre; Rosa26-LSL-YFP*. KPC organoids were derived from PDAC that developed *in KRas*^*LSL-G12D/WT*^*; P53* ^*LSL-R172H/WT*^*; Pdx-Cre*. Briefly, the PDAC tumor was dissected and cell dissociation was performed as described [54] with a few modifications. The dissected tumor was immediately placed in 5 ml of cold G solution and kept on ice. Following mechanical dissociation, the tissue was transferred to a 50 ml Falcon with 15 ml of collagenase V (1 mg/mL in DMEM plus 1% [v/v] penicillin/streptomycin [10,000 U/mL with no FCS]) and incubated for 20 min in a water bath at 37 °C. To inactivate the digestion, 15 ml of ice-cold G solution were added. The cell-dissociated PDAC was centrifuged at 300 × *g* for 5 min with low deceleration, the supernatant was discarded, and the pellet incubated in trypsin-EDTA for 5 min at room temperature. To stop the reaction, 2 ml of soybean trypsin inhibitor were added, followed by 15 ml of ice-cold G solution and centrifugation for 5 min at 300 × *g*. The pellet was resuspended in 10 ml of PBS with 2% (v/v) FCS and passed through a 70 μm filter. Cells were plated in growth factor-reduced Matrigel to grow as 3D-organoid cultures as described [55].

### Inhibitors and antibodies

The following inhibitors were used at the indicated final concentration in vitro, unless otherwise specified in the figure or figure legends: Dinaciclib (Selleck Chemicals Cat# S2768, 25 µM, 100 µM), NVP-2 (25 µM, 100 µM,), zVAD-fmk (Abcam Cat# ab120382, 20 µM). Antibodies against the following antigens were used: RNA-Pol II, pSer2 (Thermo Fisher Scientific Cat# A300-654A-M; BioLegend Cat# 920204), RNA Pol II RPB1 (BioLegend Cat# 664906), cFLIP (Enzo Life Sciences Cat# ALX-804-961-0100; AdipoGen Cat# AG-20B-0056), Caspase‐8 (Enzo Life Sciences Cat# ADI-AAM-118-E), cIAP (R and D Systems Cat# MAB3400), FADD (BD Biosciences Cat# 556402), Caspase-3 (R and D Systems Cat# AF-605-NA), Cleaved Caspase-3 (Cell Signaling Cat#9429), β-Actin (Sigma-Aldrich Cat# A5441), GAPDH (Sigma-Aldrich Cat# G9545), Bid (Cell Signaling Technology Cat# 2002), Bak (Cell Signaling Technology Cat# 3814), Bax (Cell Signaling Technology Cat# 5023), Mcl-1 (Cell Signaling Technology Cat# 5453), Bcl-2 (Santa Cruz Biotechnology Cat# sc-7382), Bcl-xL (Cell Signaling Technology Cat# 2764), XIAP (Cell Signaling Technology Cat# 2042), PARP (BD Bioscience Cat# 556362), CDK1 (Cell Signaling Technology Cat# 77055), CDK2 (Cell Signaling Technology Cat# 2546), CDK5 (Cell Signaling Technology Cat# 12134) and CDK9 (Cell Signaling Technology Cat# 2316).

### RNA interference

siRNA pools (ON-TARGET plus) containing four different siRNA sequences targeting each gene of interest were purchased from Dharmacon. Cells were transfected using Dharmafect reagent according to the manufacturer’s instructions. Cells were used for further analysis at 48 h or 72 h after transfection. Knockdown efficiency was assessed by western blotting in each experiment.

### Cell viability and cell death assays

Cell viability was determined using the Cell Titer Glo assay (Promega Cat# G7571) according to the manufacturer’s instructions. As a direct measurement of cell death, cells were seeded the day before the experiment at 10,000 cells per well of a 96 well plate. The following day cells were pretreated with the indicated inhibitors and treated with recombinant iz-huTRAIL (0.1 ng/ml-10 ng/ml) or iz-mTRAIL (1000 ng/ml) in the presence of 5 µM SYTOX® Green (Thermofisher Cat# S7020). Dead cells were imaged in real-time for the indicated times, either in 1 h or 2 h intervals via fluorescence signals using the IncuCyte FLR. Percentage of cell death was calculated counting the number of SYTOX® Green positive cells/well.

### Clonogenic assay

To analyse long-term survival (clonogenic assay), cells were seeded into 6-well plates. The next day, cells were pre-incubated with DMSO or Dinaciclib for 1 h before iz-huTRAIL was added. After 24 h dead cells were washed away, and surviving cells were cultured for additional 6 days in fresh medium without any treatment. After 7 days, cells were washed twice with PBS, fixed with methanol/acetic acid 3:1 (vol/vol) solution for 10 min at room temperature and stained with crystal violet (0.5% in 20% of methanol).

### Dynamic BH3 profiling

Briefly, 3 × 10^5^ cells were plated in a well of 6-well plate and treated with TRAIL–CDK9i for 12 h or with the indicated chemo- or targeted therapeutics for 48 h. Cells were subsequently trypsinized and 1 × 10^6^ cells/ml were used for dynamic BH3 profiling. The Bim-derived BH3-only peptide (Ac-MRPEIWIAQELRRIGDEFNA-amide) was used to assess priming status. The Bim peptide was diluted in MEB buffer (150 mM Mannitol, 10 mM HEPEs-KOH pH 7.5, 50 mM KCL, 0.02 mM EGTA, 0.02 mM EDTA, 0.1% BSA, 5 mM Succinate) and used at concentrations of (0,) 0,1, 0,3 1 and 3 μM. Cells were diluted in MEB buffer further containing 20 mg/ml oligomycin, 50 mg/ml digitonin, 100 mM JC-1, 5 M 2-mercaptoethanol. AUC values were obtained by measuring the Relative Fluorescence of the JC-1 compound using the TECAN plate reader M1000 at an excitation of 545-560 nM and its emission at 590 nM.

### Western blot analysis

Cells were treated as indicated and then lysed in lysis buffer (30 mM Tris-HCl [pH 7.4], 150 mM NaCl, 2 mM EDTA, 2 mM KCl, 10% Glycerol, 1% Triton X-100, 1× COMPLETE protease-inhibitor cocktail (Roche Cat# 5056489001)). Proteins were separated using 4–15% Mini- or Midi-PROTEAN®-TGXTM-gels (BioRad Cat# 4561086, Cat# 5671085) with Tris/glycine/SDS running buffer. Proteins were transferred on Mini- or Midi- 0.2 µm nitrocellulose membranes (BioRad transfer packs, Cat# 1704158, Cat# 1704159) using the Trans-Blot® Turbo™ Transfer System from BioRad. Proteins were detected with antibodies as indicated. Membranes were stripped with 50 mM glycine (pH 2.3) before re-probing with other antibodies.

### Tumor challenge and treatments

Preliminary data showed that a group size ≥5 was sufficient to reach statistical significance. More mice were however included and reliably reproduced the same results. Mice were randomized into treatment groups according to either the tumor photon flux value or tumor size (mm^3^).

#### A549 lung tumors

Six- to eight-week-old female Fox Chase® SCID Beige Mice (Charles River) were injected with 2 × 10^6^ A549-luc cells via the lateral tail vein. After 1 week, all mice were imaged for bioluminescence using the Ivis Spectrum (Caliper Life Science). Photons per second (Photon Flux) were quantified using the Xenogen software quantifying photons per second in a defined region of interest (ROI). Mice with established tumor burden were included in the study and randomized into the treatment groups. Subsequently, mice were treated for 4 consecutive days with daily i.p. injections of 600 µg Dinaciclib (30 mg/kg) and/or 100 µg iz-huTRAIL (5 mg/kg) or 200 µl buffer as control.

#### KP cancer model

Six- to eight-week-old *Rag1*-deficient mice of both genders were challenged intradermally (i.d.) with 5 × 10^6^ KP tumor cells on day 0. Tumors were left to develop for one week. Using a digital caliper, tumor volume was determined as: tumor volume [mm^3^] = ((length^2) *width)/2. When tumors reached a size of ~50 mm^3^, mice were randomized into treatment groups. Subsequently, mice were treated every second day for two consecutive weeks with i.p. injections of Dinaciclib (30 mg/kg) and/or iz-mTRAIL (5 mg/kg) or buffer as control in a total volume of 200 µl. Mice were humanely killed when tumors reached a size of 1.5 cm^3^. Mouse body weight was monitored during all experiments. For AST quantification, 30 µl of serum samples diluted 1:1 in PBS were used to determine AST values using Reflotron GOT test strips according to the manufacturer’s instructions.

#### Pancreatic organoid cancer model

six- to eight-week-old male C57BL/6 mice were injected subcutaneously in the flank with 1 × 10^6^ KPC cells in organoids resuspended in 100 μl of matrigel. After 1 week, tumor volume was determined as described in the previous paragraph, and tumor-bearing mice were randomized into treatment groups and treated every second day for three consecutive weeks with i.p. injections of Dinaciclib (30 mg/kg) or NVP-2 (5 mg/kg) and/or iz-mTRAIL (5 mg/kg) or buffer as control in a total volume of 200 µl. Mice were humanely killed when tumors reached a size of 1.5 cm^3^.

In order to minimize bias, all the animal experiments were conducted blindly. Technical staff made all the decisions relating to animal welfare independently of investigators. Tumor burden was evaluated by a scientist who had no indication as to which cages received which treatments.

### Study approval: animal welfare

All mice were maintained in individually ventilated cages (IVCs), *Scid-beige* mice received autoclaved food, water and bedding according to institutional guidelines under appropriate animal licenses. All animal experiments were conducted under an appropriate animal project license approved by the UK Home Office, in accordance with the revised (2013) Animals (Scientific Procedures) Act (ASPA) and the institutional guidelines of the UCL Cancer Institute, UK. The required risk assessments were obtained for this study.

### Patients and tissue samples

Tissue specimens of patients diagnosed with colorectal cancer (CRC) who underwent surgery between January 1999 and October 2014 at the Department of General and Visceral Surgery of the University Hospital Ulm were included in the study. Solely patients with adenocarcinoma were included in the study, patients with neoadjuvant therapy or tumor relapse were excluded. The comparison of CDK9 expression comprises tumor and normal tissue from 20 randomly chosen CRC patients. Samples were collected immediately during surgical resection in accordance with informed consent obtained from all patients. The study was performed with the permission of the independent local ethics committee of the University of Ulm (approvals 268/2008 and 235/2015).

### Histological quantification and immunohistochemistry (IHC)

Specimens were fixed overnight in 10% formalin, transferred to 70% ethanol 24 h later. Paraffin embedding, preparation of sections and H&E stainings were performed as part of a histological staining service at the National Heart & Lung Institute. Lung tumor burden was quantified as percentage of tumor tissue in the lung. H&E stainings were examined and quantified by an experienced pathologist (M. A. El-Bahrawy) who was blinded to the study.

For IHC, 4-µm-thick sections were de-waxed and rehydrated by passing the slides through xylene and descending grades of alcohol then rinsed in water. Slides were subjected to heat-mediated antigen retrieval by incubation in 10 mM sodium citrate (pH 6.2) at 95 °C for 15 min. Endogenous peroxidase was inactivated with 1.6% H_2_O_2_ for 10 min, followed by epitope blocking for 1 h at room temperature with 1% bovine serum albumin (Sigma-Aldrich Cat# A7030), 5% normal donkey serum (EMD-Millipore Cat# S30), and 0.4% Triton X-100 (Sigma-Aldrich Cat# 9002-93-1) diluted in PBS. Primary antibodies were incubated overnight at 4 °C, followed by secondary incubation with biotin-conjugated antibodies for 45 min at room temperature or fluorescently conjugated antibodies for 2 h. IHC signal was obtained with the avidin-biotin complex (ABC) solution (Vectastain ABC kit, Cat# LS-J1018) and developed with peroxidase substrate kit 3,3ʹ -diaminobenzidine (DAB) from Vector (Cat# SK-4100). IHC slides were counterstained with Mayer’s hematoxylin. Antibodies used included the following: Ck19 (DSHB Cat# TROMA-III).

In order to analyze the CDK9 expression in normal and colon cancer tissue, the respective tissues were initially deparaffinized in xylol and rehydrated via graded ethanol. The slices were heated in citrate buffer pH 6.0 (BioGenex Cat# C7915-010) in the microwave (450 W for 15 min followed by 270 W for 10 min) using a pressure cooker. The slices were blocked with peroxidase solution (DAKO Cat# S202386-2). and the CDK9 antibody (Cell Signaling Technology Cat# 2316) was diluted 1:150. Simple Stain N-Histofine® MAX PO rabbit (Nichirei Corporation Cat# 414141 F) was used for antigen detection. Visualization was achieved by the use of DAB from (Vector Cat# SK-4100). For counterstaining, Mayer’s hemalaun (Merck Cat# 109249) was used.

### Alcian blue/periodic acid-Schiff

To detect mucinous structures, alcian blue/periodic acid-Schiff (AB/PAS) (Abcam Cat# ab150680) staining was performed according to the manufacturer’s instructions.

### CDK9 protein expression analysis in NSCLC patients

CDK9 protein intensity data was obtained from [56]. A chemical proteomic approach was employed to capture kinases with Kinobeads (γ-version) and to quantify their abundance with label-free mass spectrometry across paired normal and tumor tissue from 14 NSCLC patients. Two technical replicates acquired for each sample were averaged and used for differential expression analysis with a paired *t* test.

### Statistical analysis

Data were analyzed using GraphPad Prism 7 software (GraphPad Prism). Results are expressed as means ± SEM. Statistical significance between groups was determined using Student’s *t* test and/or one-way analysis of variance (ANOVA), followed by the Bonferroni post-test. A *p* value of <0.05 was considered significant and indicated with *, *p* < 0.01 = ** and *p* < 0.001 = ***. ns = non-significant. Statistical significance between groups was determined using Wilcoxon signed-rank test for group comparisons demonstrated by boxplot.

## Supplementary information

Supplemental Material

## Data Availability

The proteomics dataset analyzed during the current study was downloaded from ProteomicsDB (www.proteomicsdb.org) with the data set identifier PRDB004257 [56]. No new datasets were generated during the current study.
