# Supplementary material for: Potent pro-apoptotic combination therapy is highly effective in a broad range of cancers
Source: Cell Death Differ. 2021 Sep 17;29(3):492–503. doi: 10.1038/s41418-021-00869-x (PMC8901660; doi:10.1038/s41418-021-00869-x)

Supplementary Information for

Potent pro-apoptotic combination therapy is highly effective in a broad range of cancers

Antonella Montinaro[1*], Itziar Areso Zubiaur[1*], Julia Saggau[2,3], Anna-Laura Kretz[4], Rute M.M. Ferreira[1], Omar Hassan[2,3], Ella Kitzig[4], Ines Müller[5], Mona A. El-Bahrawy[6], Silvia von Karstedt[1,2,7,8], Dagmar Kulms[5], Gianmaria Liccardi[3], Johannes Lemke[4] and Henning Walczak[1,2,3,9]

[1]Centre for Cell Death, Cancer, and Inflammation (CCCI), UCL Cancer Institute, University College London, 72 Huntley Street, London WC1E 6DD, UK

[2]CECAD Cluster of Excellence, University of Cologne, 50931 Cologne, Germany.

[3]Institute of Biochemistry I, Medical Faculty, Joseph-Stelzmann-Str. 44, University of Cologne, 50931 Cologne, Germany

[4]Department of General and Visceral Surgery, Ulm University Hospital, Albert-Einstein-Allee 23, 89081 Ulm, Germany.

[5]Experimental Dermatology, Department of Dermatology, Technical University Dresden, Dresden, Germany

[6]Department of Histopathology, Imperial College London, London W12 0NN, UK

[7]Department of Translational Genomics, Center of Integrated Oncology Cologne-Bonn, Medical Faculty, University of Cologne, 50931 Cologne, Germany

[8]Center for Molecular Medicine Cologne, Medical Faculty, University Hospital of Cologne, 50931 Cologne, Germany.

[9]Correspondence to: Henning Walczak PhD, UCL Cancer Institute & Institute of Biochemistry I, University of Cologne; Email: h.walczak@ucl.ac.uk, Phone: +44 759 025 0679 or h.walczak@uni-koeln.de, Phone: +49 221 478 81966

*These authors contributed equally to this study.

The file includes Supplementary Figures 1 to 6.

Supplementary Figure 1. The CDK9-inhibitory activity of dinaciclib and NVP-2 is required and sufficient for TRAIL sensitization. a A549 cells were transiently transfected with the indicated siRNAs. After 48 hr, transfected cells were stimulated with iz-huTRAIL at different concentrations. Cell death was measured as a function of time by Sytox Green positivity. The mean of four technical replicates of Sytox positive cells/well of one representative out of three independent experiments is shown. Representative images of indicated measurements are depicted with the corresponding percentage of dead cells. Scale bars, 200 µm. Representative western blots of knockdown efficiency are shown. **b** A549 cells were treated with NVP-2 (25 nM) or dinaciclib (25 nM) for the indicated times. Cells were lysed and subjected to western blotting. One representative of two independent experiments is shown. **c** Panel of human carcinoma cell lines. **d** Human carcinoma cell lines were treated with DMSO or dinaciclib (25nM) for 1 hr before iz-huTRAIL was added at the indicated concentrations. Cell viability was determined by CellTiter-Glo after 24 hr. Data are mean ± SEM, $n \geq 3$.**e** CDK9 protein intensity data of lung cancer patients quantified by label-free mass spectrometry across paired normal and tumor tissue. **f** Human carcinoma cell lines were treated for up to 48 hr with DMSO, carboplatin (10 µM), cisplatin (10 µM), 5-FU (10 µM), paclitaxel (10 µM), oxaliplatin (10 µM), gemcitabine (1 µM), dinaciclib (25 nM) and iz-huTRAIL(10 ng/ml) cell viability was determined by CellTiter-Glo.

Supplementary Figure 2. Randomization of KPC tumor-bearing mice. Tumor volume one week after subcutaneous injection of 1 x 10^6 KPC cells.

Supplementary Figure 3. Randomization of tumor-bearing mice and sensitivity of NSCLC cells to TRAIL-CDK9i combination. a KP cells were treated with DMSO or dinaciclib (100 nM) for 1 hr before iz-mTRAIL was added at the indicated concentrations. Cell viability was determined by CellTiter-Glo after 48 hr. Data are mean ± SEM, $n \geq 3$. **b** Randomization of KP tumor-bearing mice: tumor volume one week after intradermal injection of 0.5 x 10^6 KP cells. **c** Tumor burden quantification by bioluminescence imaging (Photon Flux) one week after injection. Dots represent individual mice (n=11 per group) ± SEM before treatment (after randomization). **d** Histological quantification of tumor

burden as the percentage of total lung area occupied by tumor tissue. Representative images of H&E-stained lung sections (5x magnification) are shown.

Supplementary Figure 4. Resistance to chemo- and targeted therapy. a Gemcitabine-sensitive and -resistant pancreatic cancer cells were treated with gemcitabine at the indicated concentrations. Cell viability was quantified after 48 hr. **b** Sorafenib-sensitive and -resistant hepatocellular carcinoma cells were treated with sorafenib at the indicated concentrations of. Cell viability was quantified after 24 hr. **c** Mutations in BRAF, NRAS and/or p53 of cell lines included in the melanoma panel are indicated. Sensitive cells were treated in the presence of the indicated drugs for six months which rendered the resistant variants of the respective melanoma cell lines. **d** Cell viability of sensitive and resistant melanoma cells treated for 48 hr with dabrafenib (10 µM), trametinib (10 nM), cisplatin (10 µM) or DMSO as control. Data are mean ± SEM, $n \geq 3$.

Supplementary Figure 5. Dynamic BH3 profiling of drug-sensitive- and drug-resistant melanoma cells. a Trametinib-sensitive and -resistant SKmel2 **b**, Dabrafenib-sensitive and -resistant Malme3M, **c** cisplatin-sensitive and -resistant IGR-37 cells were treated for 48 hr with dabrafenib (10 µM), trametinib (10 nM) or cisplatin (10 µM), respectively, or for 12 hr with a combination of dinaciclib (25 nM) and TRAIL (10 ng/ml). Cells were subsequently permeabilized, stained with the fluorescent dye JC-1 and incubated in the absence or presence of the Bim-derived BH3-only peptide employed at different concentrations (0.1 – 3.0 µM) to induce mitochondrial depolarization and MOMP. Results are expressed as Δ% priming (increase in priming compared to untreated cells, left panels. Relative Fluorescent Unit at 590 nm average and standard deviation from triplicated wells of a 384-well plate (left panels).

Supplementary Figure 6. Concomitant downregulation of cFLIP and Mcl-1 in both, drug-sensitive and -resistant melanoma cells. a Dabrafenib-sensitive and -resistant Malme3M, **b** cisplatin-sensitive and -resistant IGR-37 cells were treated with dabrafenib (10 µM), trametinib (10 nM) or cisplatin (10 µM), respectively, or with dinaciclib (25 nM) for the indicated times. Cells were lysed and

subjected to western blotting with antibodies specific for the indicated antigens. One representative of two independent experiments is shown. * indicates an unspecific band.

Supplementary Fig. 1

a

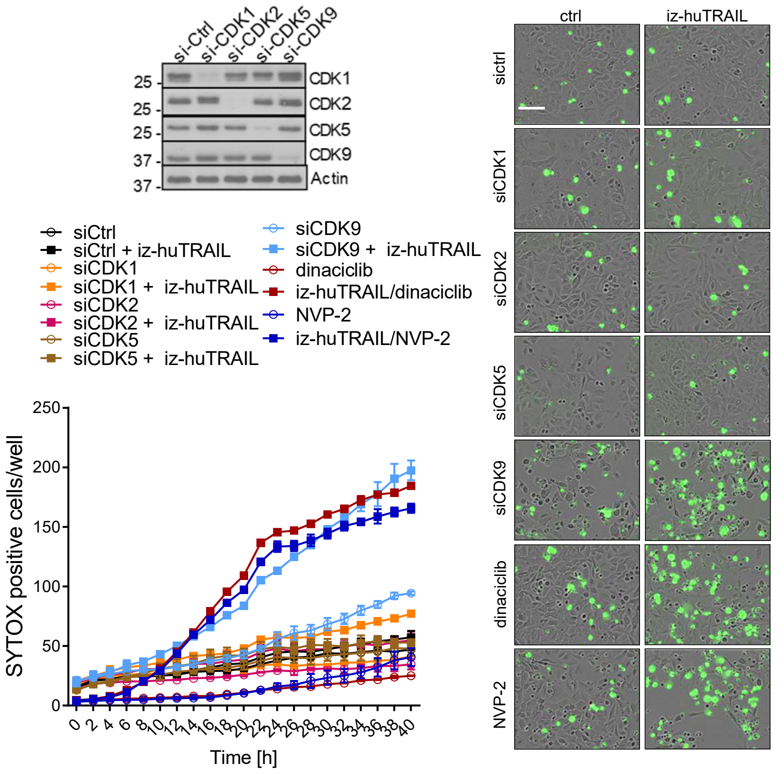

b

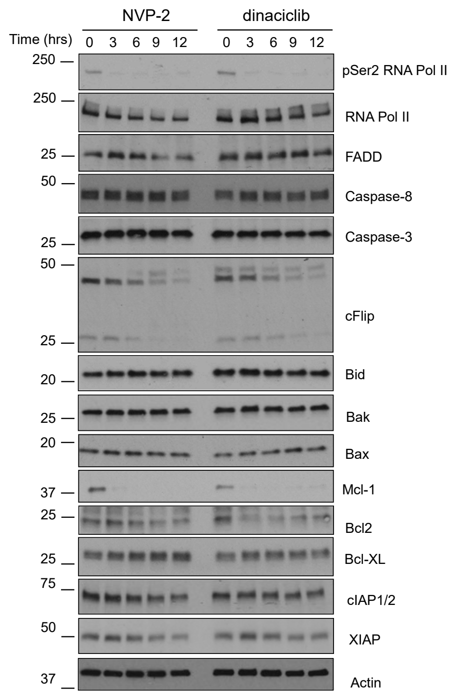

c

Tissue of Origin	Cell lines
Lung	A549
	H460
	H320
	Calu-1
Cervix	HeLa
Breast	MDA-MB 231
Ovarian	PEA1
	PE04
Colon	HCT-116
Melanoma	SKmel2
Liver	JHH4
	Hep3B
Pancreas	Colo 357
	A818-6
	BxPC-3

d

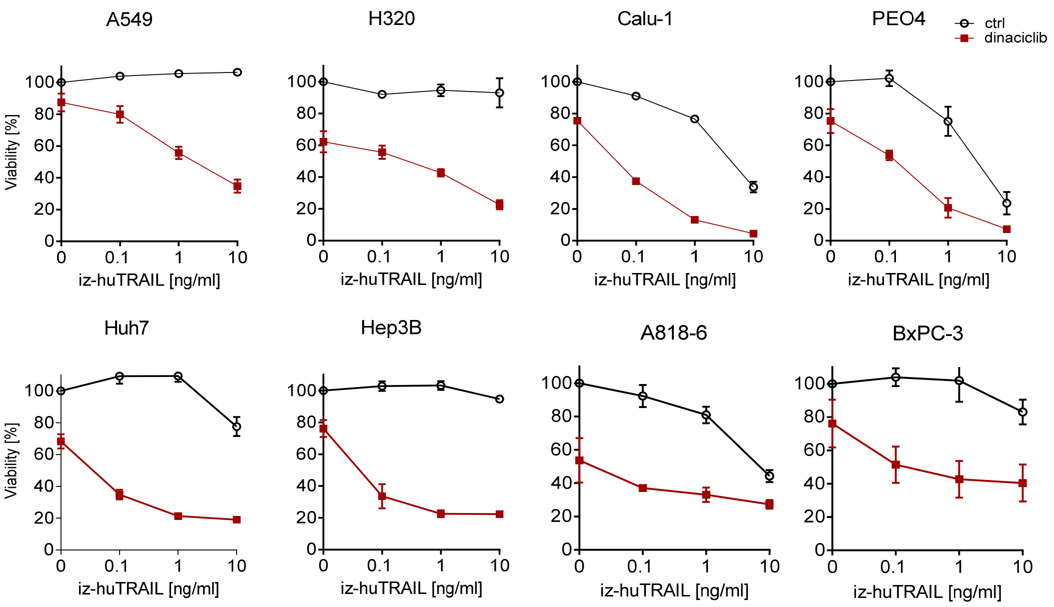

e

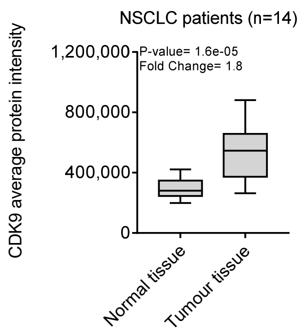

f

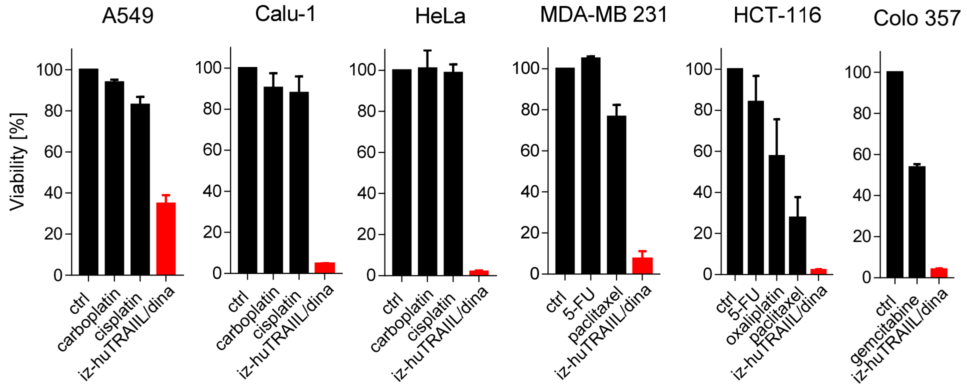

Supplementary Fig. 2

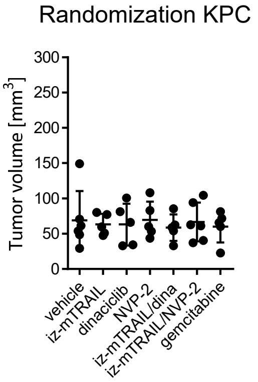

Supplementary Fig. 3

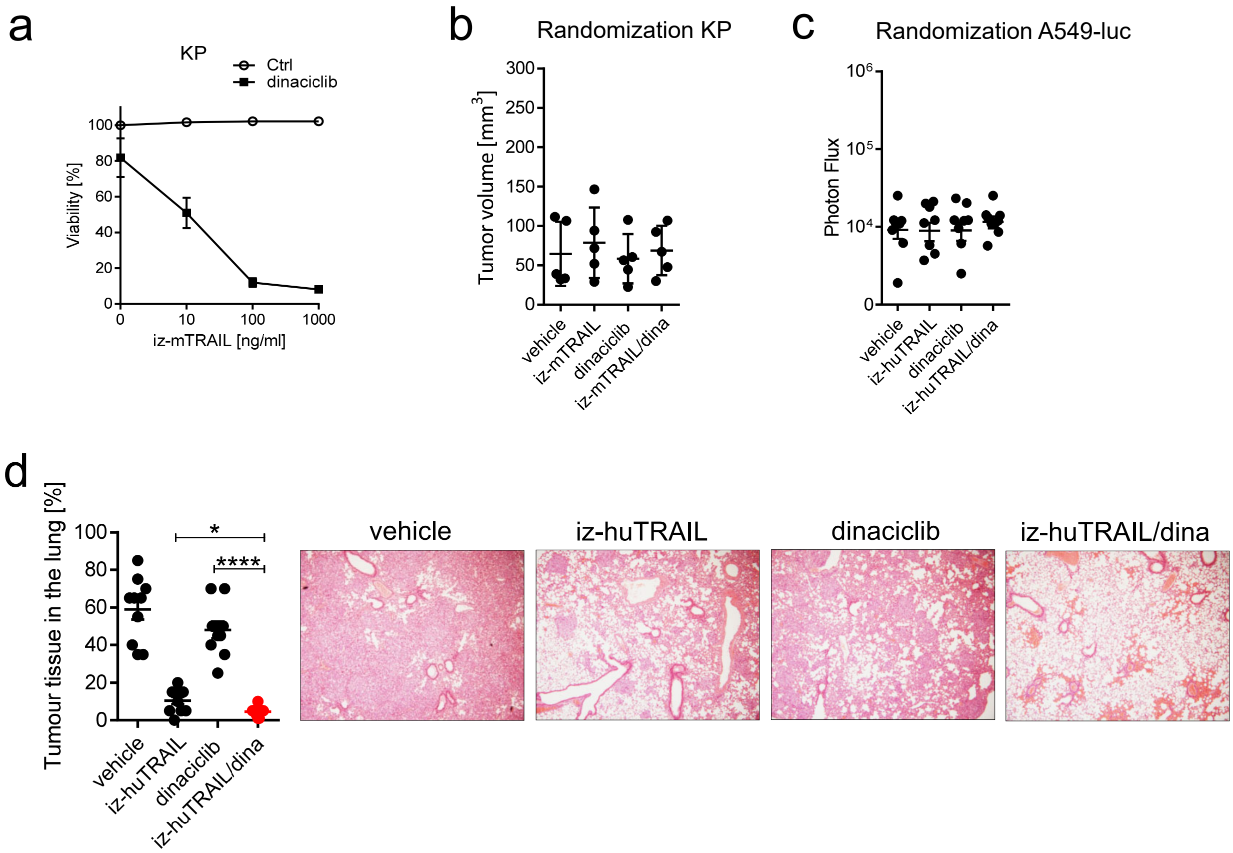

Supplementary Fig. 4

a

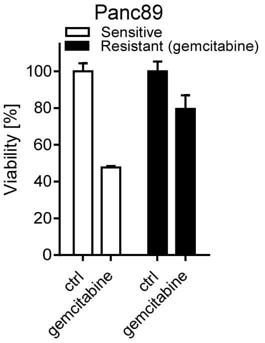

b

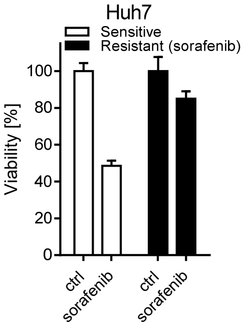

c

Cell line	BRAFmut	NRASmut	p53mut	Resistant to
Malme3M	mut	wt		
Malme3M	mut	wt		dabrafenib
WM3248	mut	wt	mut	
WM3248	mut	wt	mut	dabrafenib
SKmel2	wt	mut		
SKmel2	wt	mut		trametinib
SKmel147	wt	mut		
SKmel147	wt	mut		trametinib
IGR-37	mut	wt		
IGR-37	mut	wt		cisplatin
A375	mut	wt		
A375	mut	wt		cisplatin

d

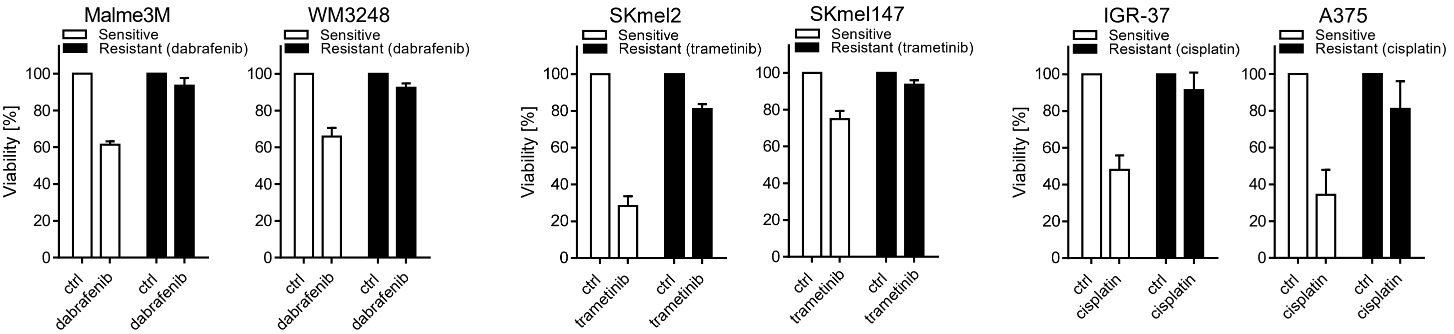

Supplementary Fig. 5

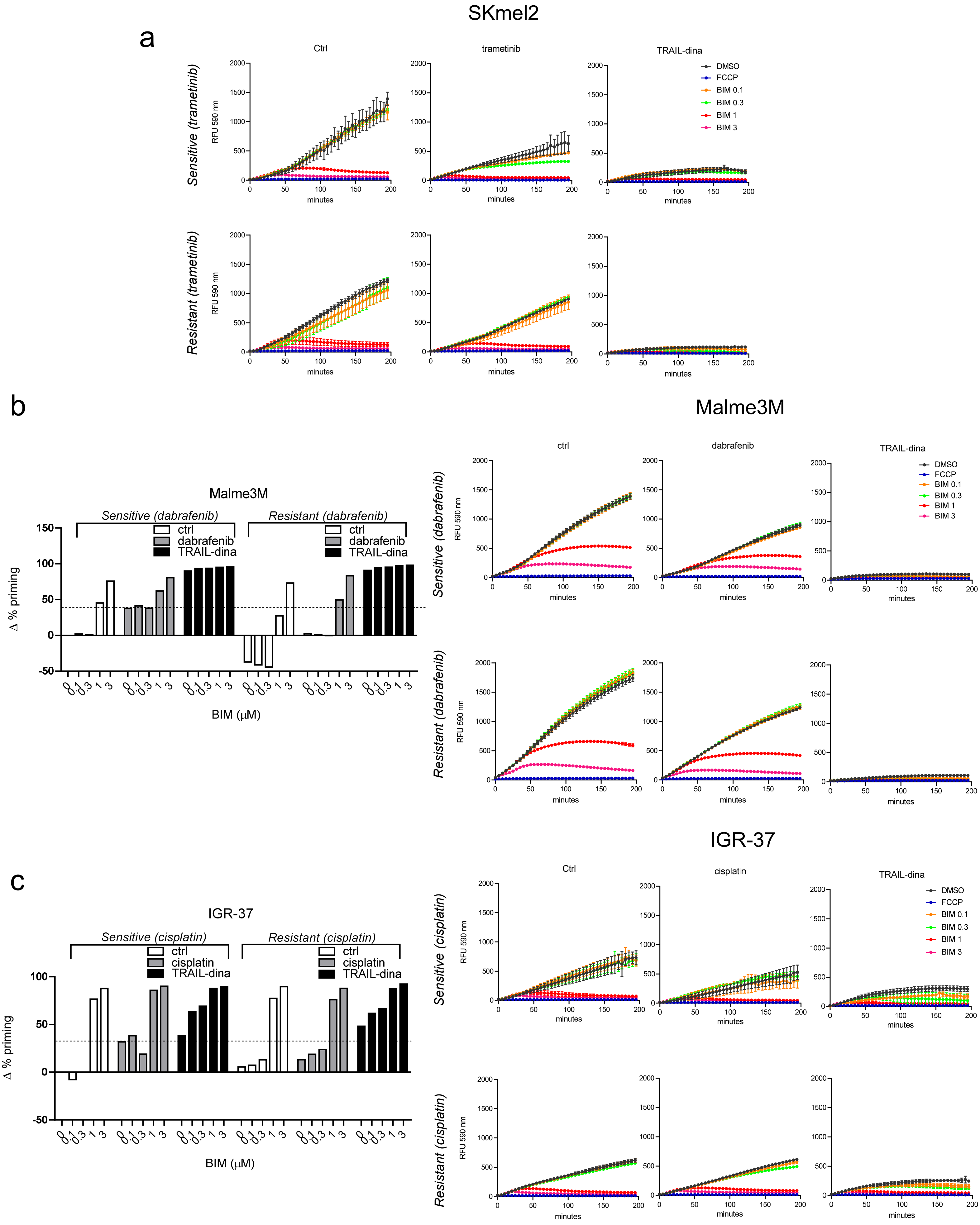

Supplementary Fig. 6

a

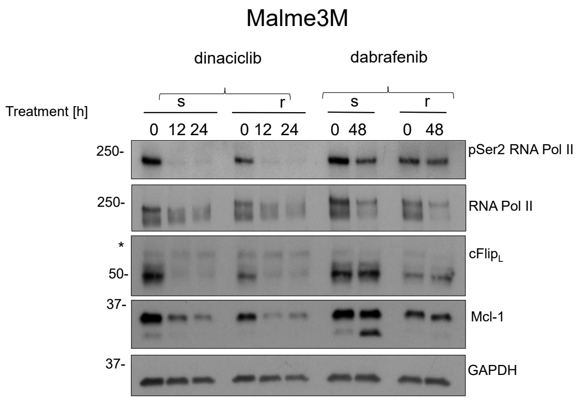

b

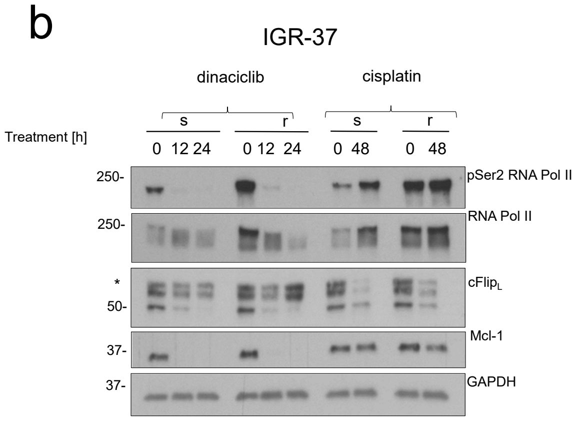

Supplement: Supplementary file 1 — Supplemental Material [file 41418_2021_869_MOESM1_ESM.pdf]
